# Food-Predicting Stimuli Differentially Influence Eye Movements and Goal-Directed Behavior in Normal-Weight, Overweight, and Obese Individuals

**DOI:** 10.3389/fpsyt.2017.00230

**Published:** 2017-11-13

**Authors:** Rea Lehner, Joshua H. Balsters, Alexandra Bürgler, Todd A. Hare, Nicole Wenderoth

**Affiliations:** ^1^Neural Control of Movement Laboratory, Department of Health Science and Technology, ETH Zurich, Zurich, Switzerland; ^2^Neuroscience Center Zurich, ETH Zurich, University of Zurich, University and Balgrist Hospital Zurich, Zurich, Switzerland; ^3^Department of Psychology, Royal Holloway University of London, Egham, United Kingdom; ^4^Laboratory for Social and Neural Systems Research, Department of Economics, University of Zurich, Zurich, Switzerland; ^5^Movement Control and Neuroplasticity Research Group, Department of Kinesiology, Biomedical Sciences Group, KU Leuven, Leuven, Belgium

**Keywords:** Pavlovian-to-instrumental transfer, cue-controlled behavior, incentive salience, conditioned response, eye movements, obesity

## Abstract

Obese individuals have been shown to exhibit abnormal sensitivity to rewards and reward-predicting cues as for example food-associated cues frequently used in advertisements. It has also been shown that food-associated cues can increase goal-directed behavior but it is currently unknown, whether this effect differs between normal-weight, overweight, and obese individuals. Here, we investigate this question by using a Pavlovian-to-instrumental transfer (PIT) task in normal-weight (*N* = 20), overweight (*N* = 17), and obese (*N* = 17) individuals. Furthermore, we applied eye tracking during Pavlovian conditioning to measure the participants’ conditioned response as a proxy of the incentive salience of the predicted reward. Our results show that the goal-directed behavior of overweight individuals was more strongly influenced by food-predicting cues (i.e., stronger PIT effect) than that of normal-weight and obese individuals (*p* < 0.001). The weight groups were matched for age, gender, education, and parental education. Eye movements during Pavlovian conditioning also differed between weight categories (*p* < 0.05) and were used to categorize individuals based on their fixation style into “high eye index” versus “low eye index” as well. Our main finding was that the fixation style exhibited a complex interaction with the weight category. Furthermore, we found that normal-weight individuals of the group “high eye index” had higher body mass index within the healthy range than individuals of the group “low eye index” (*p* < 0.001), but this relationship was not found within in the overweight or obese groups (*p* > 0.646). Our findings are largely consistent with the incentive sensitization theory predicting that overweight individuals are more susceptible to food-related cues than normal-weight controls. However, this hypersensitivity might be reduced in obese individuals, possibly due to habitual/compulsive overeating or differences in reward valuation.

## Introduction

The worldwide increase of individuals being overweight or obese produces a high medical and psychosocial burden (1–4), particularly since this condition is related to several comorbidities, such as cardiovascular disease, which is known as the global leading cause of death (2, 4).

One factor which has been hypothesized to influence decision-making in the context of ingestive behavior and energy balance (5, 6) is the augmented food marketing (7–10) creating a so-called “obesogenic” environment, i.e., customers are surrounded by a plethora of food-associated sensory cues reminding them constantly of meals or drinks as for example food packaging images at train stations, Coke commercials on TV, or the two arches of the McDonald’s sign in front of every store.

Recent studies in humans have shown that food-associated cues influence behavior even when satiated or when rewards are no longer available (11–14). Initial reward-seeking behavior controlled by food cues might lead to habitual and ultimately compulsive overeating as suggested by the incentive sensitization theory of addiction (15–21). The theory implies that in a first phase, motivational value is directed to the reward itself, and in a second phase, to the cues and objects related to the reward, turning them into attention-grabbing incentives (20). In animals, this process can be measured by the Pavlovian conditioned approach/response, i.e., when animals start to sniff, lick, or bite the lever or food tray, which predicted reward delivery (22–24). Such cues can then become motivators and act as reinforcers themselves leading to strong reward-seeking behavior (15, 25, 26). However, it is currently controversially debated whether this model developed in the context of addiction applies also to obesity (6, 19, 20, 27–31). Previous studies have shown an abnormal sensitivity to rewards and reward-predicting cues in obese individuals (32–38) but did not test whether this modulates goal-directed behavior. Here, we address this question and investigate whether food-predicting cues differentially influence goal-directed behavior of normal-weight, overweight, and obese individuals. We employed Pavlovian-to-instrumental transfer (PIT) [for review, see Ref. (26)] to measure the influence of food-related cues on goal-directed behavior. The PIT phenomenon has been widely investigated in both animals [for review, see Ref. (25)] and humans (11–13, 26, 39–57), making this a useful paradigm for translational research.

Furthermore, we applied eye tracking during Pavlovian conditioning as a proxy of the incentive salience of the predicted reward, which might explain potential individual differences. Several studies in rodents have shown that there is considerable individual variation when the extent to which individuals attribute motivation to reward-predicting cues was estimated (22, 23, 58–62). However, it is currently unclear how these findings from animal research translate to humans since the only two available studies (49, 63) substantially differed in how conditioned responses were defined and quantified.

## Materials and Methods

### Participants

In total, 64 volunteers were recruited for this case–control study. The following recruitment strategies were used: announcements of the Swiss Adiposity foundation and advertisements in local clinics, self-help groups, plus-size clothing stores and on the university website. Participants were included when they complied with the following criteria: age 18–65 years, German speakers, normal or corrected-to-normal vision with contact lenses and no food allergies against any ingredient of the four foods used in the experiment (i.e., Maltesers chocolate, Haribo gummy bears, TUC crackers, and Zweifel crisps).

Participants with a diagnosis of any psychological or neurological disease, drug abuse in the past, ocular problems, or intake of psychiatric or neuroleptic drugs during the last 6 months were excluded (i.e., three participants). Five additional participants were excluded because they failed to learn the instrumental and/or the Pavlovian associations. We used the body mass index (BMI) classification according to the World Health Organization (64), to differentiate between normal-weight (BMI < 25 kg/m^2^), overweight (25 kg/m^2^ ≥ BMI < 30 kg/m^2^), and obese individuals (BMI ≥ 30 kg/m^2^). BMI was calculated by dividing the individual’s weight (kilograms) by the square of the individual’s height (meters). Weight was measured on a flat scale (Seca 635, Seca, Hamburg, Germany) and height with a mechanical telescopic measuring rod (Seca 222, Seca, Hamburg, Germany). To take into account that a high BMI can arise due to high muscle mass, the participants with a BMI ≥ 25 were asked to estimate if this was due to increased muscle or fat mass. Selecting the muscle mass option led to exclusion (i.e., two participants). The final sample included fifty-four participants (mean age = 31 ± 10 years, mean ± SD, oldest participant = 55 years, 55.6% female). Although the age range of our sample was broad, changes in coping strategies and comorbidities over one’s lifetime should not have confounded our results due to group matching. Cases and controls were matched for age, gender, education, and parental education. The final sample characteristics are shown in Table 1.

**Table 1:** Descriptive statistics (mean ± SD) for each weight category based on body mass index (BMI).

		Normal-weight (*N* = 20)	Overweight (*N* = 17)	Obese (*N* = 17)	*p*-Value
Age (years)		29 ± 9	30 ± 8	33 ± 12	0.553
Gender	Male	7	11	6	0.127
	Female	13	6	11	
Education		2 ± 0	2 ± 0	2 ± 1	0.363
Parental education		1.5 ± 0.4	1.5 ± 0.5	1.2 ± 0.4	0.170
Barratt Impulsiveness Scale	Non-planning	11 ± 2	10 ± 3	11 ± 3	0.843
	Motor	13 ± 3	13 ± 2	13 ± 3	0.957
	Attentional	11 ± 2	11 ± 3	10 ± 2	0.727
Depression (Beck Depression Inventory)		4 ± 3	7 ± 7	7 ± 6	0.342
Food liking		7.91 ± 1.70	7.51 ± 1.03	7.78 ± 1.36	0.281
Perception of neutral outcome		3.0 ± 2.7	3.2 ± 2.9	1.8 ± 1.8	0.463
Waist circumference (cm)	Male	84.1 ± 6.9	96.5 ± 5.3	109.7 ± 12.9	0.001
	Female	72.9 ± 3.0	91.7 ± 9.9	110.4 ± 15.3	
BMI (kg/m^2^)		21.9 ± 1.6	27.3 ± 1.5	36.9 ± 5.1	0.001

*No significant differences were found in age, gender, education, or parental education (Chi-squared/Kruskal–Wallis test). For education, the higher the value, the higher the education. No significant differences were found in impulsiveness scores, total depression score, food liking and perception of neutral outcome (ANOVA/Kruskal–Wallis test). As expected, waist circumference and BMI differed significantly between weight categories*.

All subjects gave written informed consent in accordance with the Declaration of Helsinki. The protocol was approved by the Ethics Committee of the Canton Zurich. Participants were reimbursed with 20 Swiss francs per hour and a snack (i.e., a package of the chosen food and an apple).

### Indirect Measures for Body Fat: BMI and Waist Circumference

Overeating high-calorie and palatable foods mainly leads to the accumulation of visceral fat (65), which is reflected in waist circumference measurements (see Table 1). Waist circumference was measured on the approximate midline between the top of the pelvis bone and the lower margin of the most caudal palpable rip. It was measured by holding the measuring tape horizontally to the floor (64, 66).

### Questionnaires

All participants completed a number of questionnaires in German (see Table 1). The following personal details were retrieved: gender, date of birth, education of the participant, as well as parental education. Participants filled in a standard handedness questionnaire (67) to determine the dominant hand for making button presses during the tasks.

We included a measure of self-reported impulsiveness by means of the short 15-item version of the Barratt Impulsiveness Scale [BIS; (68–70)]. The BIS has good internal consistency and test-retest reliability (71). It differentiates between three subscales of impulsiveness: non-planning, motor and attentional impulsiveness.

We measured self-reported depression symptoms by means of the 21-item version of the Beck Depression Inventory [BDI-II; (72–74)]. The BDI-II shows a high internal consistency and test–retest reliability (74).

Furthermore, the preferred snack out of four different options was assessed. Four palatable, high-calorie snacks were used because it was previously shown that the PIT effect was stronger for these food products (14). Our selection included two sweet ones, pieces of chocolate and gummy bears, and two savory ones, crackers, and crisps. In a first step, participants had to rate them according to how much they liked them (1 = I like it best, 4 = I like it least). In a second step, a visual analog scale was used to quantify how much they liked their first choice. A picture of the participant’s choice was subsequently utilized as reward/outcome in the PIT experiment.

After the instrumental and Pavlovian conditioning task, participants answered a query to check if they learned the correct associations (i.e., response–outcome in instrumental conditioning, stimulus–outcome in Pavlovian conditioning). At the end of the learning phase, participants rated how they perceived the neutral outcome on a visual analog scale (0 = neutral, 10 = punishment).

There were no significant differences between weight groups for impulsiveness, depression symptoms, food liking and perception of the neutral outcome between the three weight groups (ANOVA/Kruskal–Wallis test, Table 1).

### Behavioral Experiment

#### Experimental Setup

The experimental setup consisted of an eye-tracker with the corresponding monitor (Tobii TX300 Eye Tracker, Tobii Technology, Stockholm, Sweden), a custom-made chin rest and a computer (HP EliteDesk 800 G1 Small Form Factor PC, HP Inc., Palo Alto, CA, USA).

**Figure 1:**
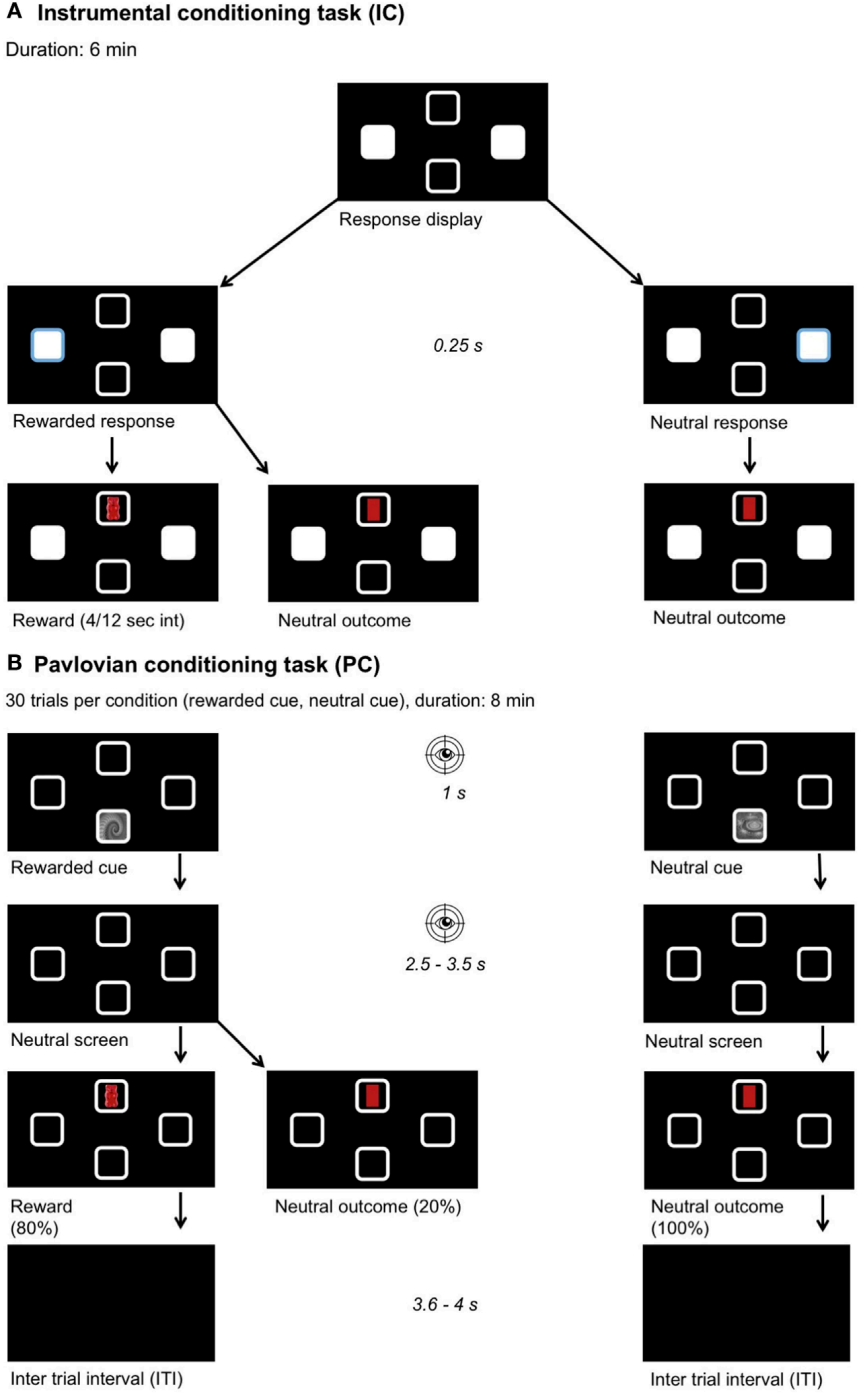

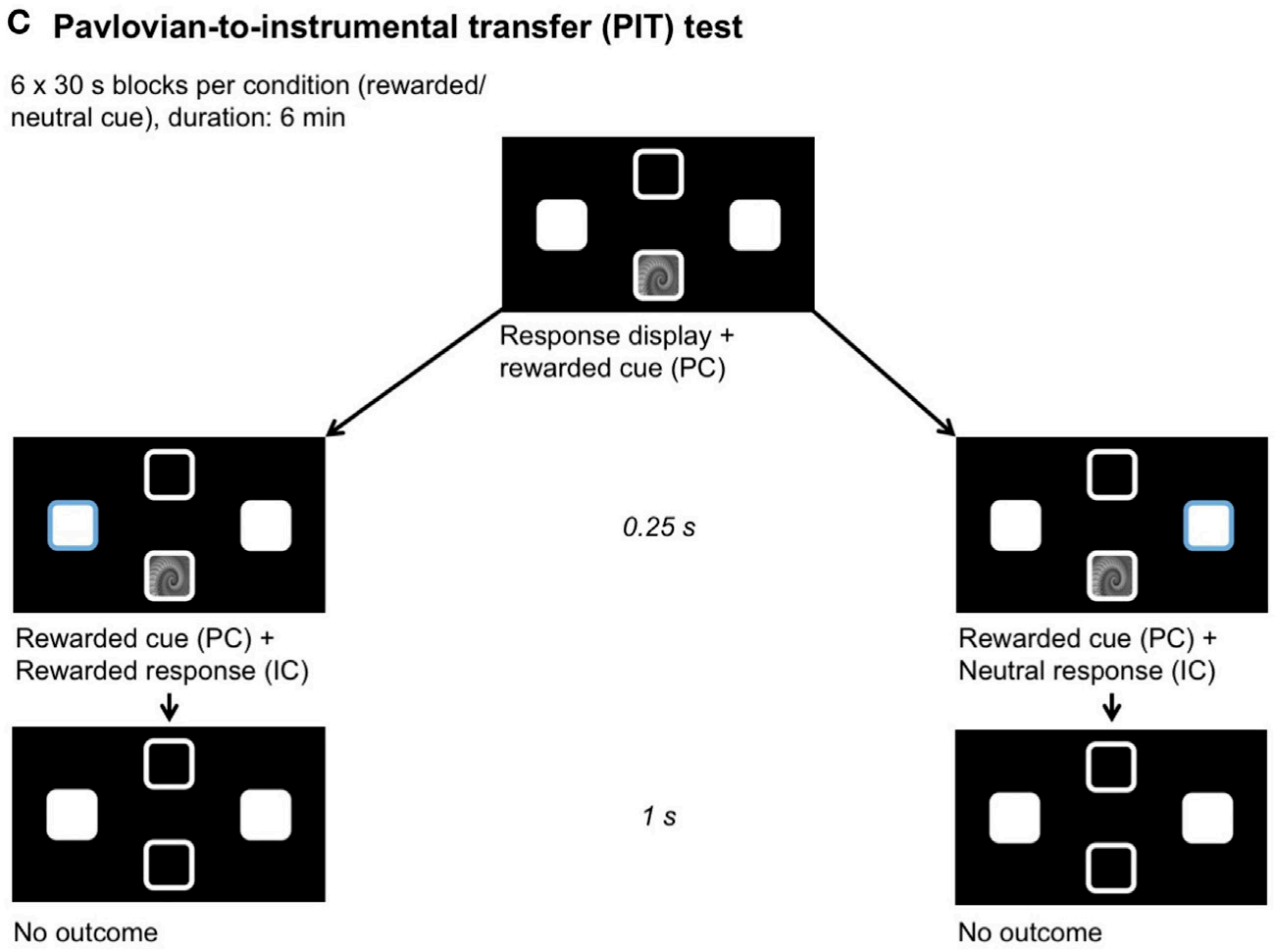
Experimental setup. Participants chose their preferred food out of four options (chocolate, gummy bears, crackers, crisps). A picture of this food was then used as a reward during learning. Participants were instructed they would receive a proportional amount of the collected foods after the experiment. The position of the outcome and stimulus and all learning associations were pseudo-randomized across participants. **(A)** Instrumental conditioning: participants learned the response–outcome associations, one key press yields a reward and the other a neutral outcome. A partial reinforcement schedule was used with a variable time interval between 4 and 12 s. **(B)** Pavlovian conditioning: participants learned the stimulus–outcome associations, one fractal yields a reward and the other a neutral outcome. After stimulus presentation, a neutral screen showing four empty squares appeared. Eye movements were recorded during stimulus and neutral screen presentation to measure the conditioned response toward the rewarded cue. **(C)** Pavlovian-to-instrumental transfer task: we measured the influence of the previously learned associations on the response behavior when the same stimuli as before were presented and under nominal extinction.

We used two gray-scaled fractals as stimuli during the Pavlovian conditioning and PIT task, which were matched for luminance and complexity (75). Furthermore, we used images of Maltesers chocolate, Haribo gummy bears, TUC crackers and Zweifel crisps on a black background as reinforcing food outcomes during instrumental and Pavlovian conditioning (Figure 1). Only the participant’s favorite food choice was used as a reinforcing outcome in the subsequent tasks. Note that participants were instructed that these images represented real food rewards, which were collected throughout the experiment and received at the end. The corresponding neutral outcome cues had a similar shape and color as the original food item (i.e., yellow oval for crisps) but without the rewarding property. Given that the visual properties of the outcomes were matched, differences in the eye movements can be narrowed down to the rewarding properties of the food outcome.

#### General Procedure

We used a standard PIT paradigm [for review, see Ref. (26)], consisting of three tasks: an instrumental conditioning task (i.e., response–outcome associations were learned), a Pavlovian conditioning task (i.e., stimulus–outcome associations were learned) and finally, a PIT test. The experiment was programmed in Matlab (version R2013b, The Mathworks Inc., Natick, MA, USA) by means of the Psychtoolbox [version 3; (76)].

Participants were asked to abstain from eating for 4 h before the experiment in order to increase the incentive value of the food and the food-related cue (60). The experiment was performed between 8 a.m. and 7.30 p.m. depending on laboratory, experimenter, and participant availability. A control analysis did not reveal an effect of time of testing on PIT (*r* = −0.08, *p* = 0.550), nor did weight groups differ in the time of testing (ANOVA, *p* = 0.208). Note that we did not control for sleep quantity or quality on the night preceding the experimental day, which can alter the incentive value of food (77) and performance on visual and cognitive tasks (78, 79). Also, we did not collect data on the phase of the menstrual cycle and thus cannot estimate or control for any effects of menstrual phase on our measures of interest. It has been shown that circulating estradiol concentrations have an influence on energy consumption (80) and may reduce food intake by decreasing neural activity to food cues in visual cortical pathways associated with reward (80, 81).

Participants received a general verbal instruction before the experiment. Before each task, three to four example trials were shown by one of two female experimenters to rule out any misunderstandings. During the tasks, the participants had to position their chin on the chin rest. They were instructed to look at the screen during the whole experiment, to maintain a stable position of their head and to blink as little as possible. Importantly, they were told that they would receive all food outcomes collected during the whole PIT experiment after the experiment. Hence, participants did not explicitly know how many rewards they collected in the instrumental and Pavlovian task, which reduces a possible satiation effect. The light was switched off during the whole experiment to improve the quality of the eye tracking and keep conditions constant over all three tasks of the PIT experiment.

##### Instrumental Conditioning Task

The goal of this task was that the participants learned the response–outcome associations (Figure 1A). The participant was free to choose between two different response options (left or right) by using their dominant hand to make a left arrow or a right arrow key press. One of these keys was assigned to the food (e.g., crisp), the other to a neutral outcome, which had a similar shape and color as the food (i.e., yellow oval). The response that lead to a reward was called the “rewarded response,” the other “neutral response.” After the response, the reward or the neutral outcome was shown for 1 s in the top or bottom square depending on the randomization. A partial reinforcement schedule was used with a variable time interval between 4 and 12 s (4/12 s interval). This means that after a rewarded response followed by rewarded outcome, the subsequent rewarded responses for a delay period of 4–12 s led to a neutral outcome. This task lasted 6 min. The participants were asked to collect as many rewards as possible and to memorize, which key was associated with the reward. Participants were told that not every “rewarded response” will lead to a reward (i.e., awareness of the partial reinforcement schedule). Directly after completing the task, the participants were tested on the response–outcome associations. In average, only 20% of all responses were rewarded.

##### Pavlovian Conditioning Task

The goal of this task was to learn the cue-outcome associations (Figure 1B). An optical eye-tracker (Tobii TX300 Eye Tracker, Tobii Technology, Stockholm, Sweden) was used for measuring eye movements. Eye movements were recorded at 60 Hz in order to analyze the amount of time spent within two areas of interest. The areas of interest were defined as the upper and lower square (8.4 cm^2^), where the cue and the outcome were presented. Eye movements in these two areas of interest (i.e., upper and lower square) were taken as a measure for the conditioned response that arises in the time course of the Pavlovian conditioning task (49). This conditioned response was later used to categorize the participants into sign- and goal-trackers. Randomly one of the two possible cues was displayed either on the top or bottom square of the screen for 1 s. One cue was associated with the food reward, called the “rewarded cue,” and the other was associated with the neutral outcome, called “neutral cue.” The cue–outcome associations were counterbalanced across participants. The outcomes were presented in the same square as during instrumental conditioning and the cues were presented in the opposite square. After stimulus presentation, a neutral screen showing the four empty squares appeared. Eye movements were recorded during cue and neutral screen presentation. This neutral screen was used because otherwise eye movements are naturally biased toward visible cues. The presentation of the neutral screen was jittered between 2.5 and 3.5 s. After the jitter, the reward or the neutral outcome contingent to the presented cue was displayed for 1 s. The rewarded cue was followed by a reward in 80% of the trials and by a neutral outcome in 20% of the trials, whereas the neutral outcome always succeeded the neutral cue (100%). The participant was told to memorize the contingencies. There was an inter-trial-interval (ITI) lasting 3.6–4 s. The ITI (mean = 3.8 s) was deliberately chosen to be longer than the jitter (mean = 3 s), in order to ensure close temporal proximity of the cue to the contingent outcome. Thirty trials per condition were performed and the whole task took about 8 min. In total, 24 rewards were acquired during this task.

##### PIT Test

The goal of this task was to measure the influence of the previously learned associations on the response behavior (Figure 1C). During the PIT test, the response display of the instrumental conditioning task together with cues from the Pavlovian conditioning were presented. In blocks of 30 s, the rewarded and neutral cue were randomly displayed in the square corresponding to the one used during Pavlovian conditioning. Again here, the participants were free to make as many responses with their dominant hand as they wanted to. The test was performed under nominal extinction meaning that their response did not lead to any displayed outcome but the participants were instructed that the rewards were counted in the background. Participants were not explicitly told to collect as many rewards as possible or to pay attention neither to ignore the Pavlovian cues. The task lasted 6 min, each cue was shown for 30 s and six times.

### Analysis

#### Eye-Tracking Data

Eye tracking of the first second of each trial (i.e., during cue presentation) was discarded because all participants fixated the cue. From the remainder, the variable “eye index” was calculated for each participant, each cue (rewarded or neutral) and for six bins of five trials of the Pavlovian conditioning task. We only considered fixation periods greater than 116 ms as suggested by previous literature (49). The eye index was calculated as the time on reward location as a percentage of the total time spent on the reward and cue location (i.e., upper and lower square):

$$\text{eye index}=\frac{\text{time on reward location}}{\text{time on reward location + time on cue location}}*100.$$

Even though most participants spent more time on the reward location, there were individual differences in how long participants looked at the cue location. Therefore, a “fixation style” was derived for each participant based on a median split of the eye index based on data from the second half (trials 16–30) of the reward condition. We used the second half of the data because contingency learning has been shown to be stable during the later phases of Pavlovian conditioning experiments (49). Individuals of the group “low eye index” looked relatively longer at the cue location than individuals of the group “high eye index.”

#### Behavioral Data

The “PIT effect” is defined as an interaction between “condition” and “response,” i.e., when participants make more rewarded than neutral responses during the presentation of the reward-predicting cue and *vice versa* for the neutral cue. The higher the PIT effect, the stronger is the influence of the Pavlovian cue on goal-directed behavior.

#### Statistics

The data were analyzed using mixed-effects models in SPSS 23 (IBM Corp., Armonk, NY, USA). Mixed-effects models are more robust to non-normal distributed data and show a better fit for repeated measurements than conventional ANOVAs (82, 83). Depending on the analysis, condition and time or condition and response were modeled as fixed effects and subjects were always modeled as a random effect. We used a compound symmetry covariance structure, which assumes nearly equal variance and covariance across factors and is, therefore, a good fit for repeated measures designs (84). Based on previous literature (49, 52, 85–87), we added impulsiveness and depression as covariates of no interest to our statistical model of PIT. Bonferroni-corrected *post hoc* tests were applied if a significant main effect was detected the linear mixed-effects models. We report Cohen’s *d* as a measure for effect size (small *d* = 0.20–0.49, medium *d* = 0.50–0.80, large *d* > 0.80) (88).

## Results

### Instrumental Task

**Figure 2:**
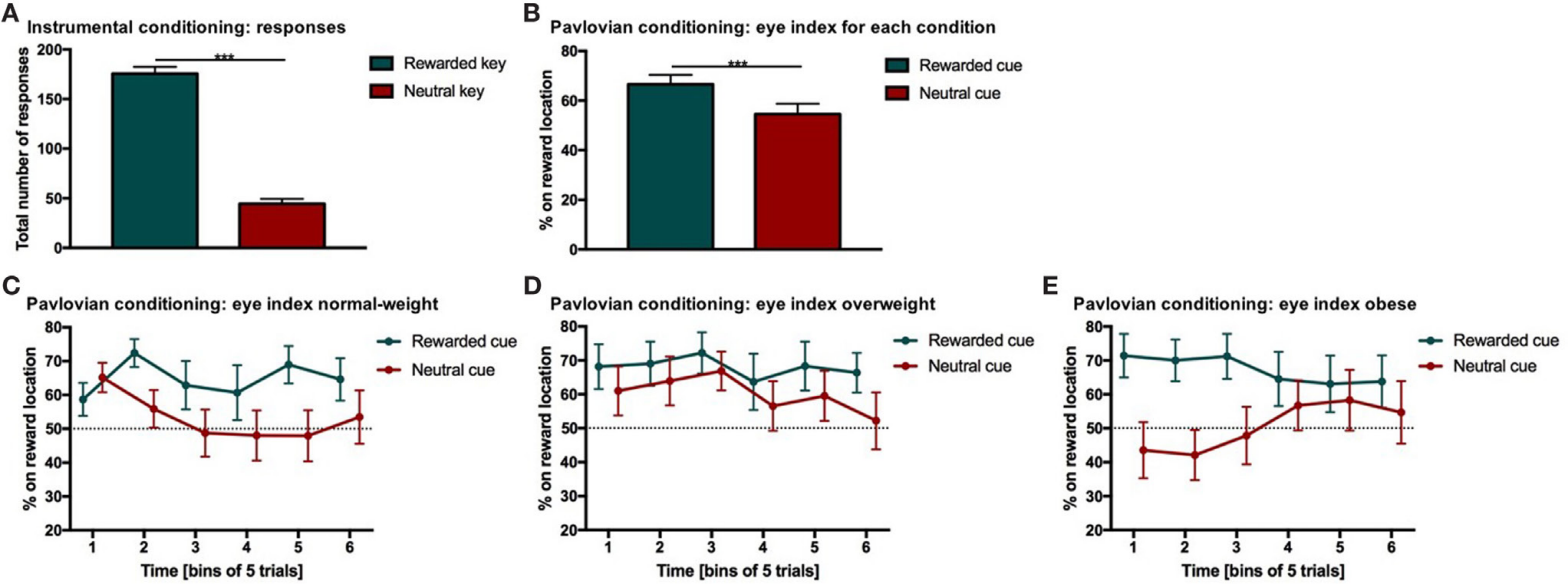
Results from instrumental and Pavlovian conditioning. Error bars indicate SEM. The rewarded key/condition is depicted in green and the neutral key/condition in red. **(A)** Total number of responses for each condition during instrumental conditioning. Participants chose the rewarded response significantly more often than the neutral response (***p_RESPONSE TYPE_ < 0.001, *d* = 2.9). **(B)** Percentage of time on reward location during Pavlovian conditioning. The eye index indicates if more time was spent on the reward location or on the cue location of the screen. The eye index was analyzed in bins of five trials for the rewarded and neutral condition and each weight category. The percentage of time on the reward location differed significantly between rewarded and neutral trials (***p_CONDITION_ < 0.001, *d* = 0.41). **(C–E)** Percentage of time on reward location during Pavlovian conditioning for each weight category. The eye index differed significantly between conditions over time and weight categories (normal-weight *N* = 20, overweight *N* = 17, obese *N* = 17, *p*_CONDITION*TIME*WEIGHT CATEGORY_ < 0.05).

Participants (*N* = 54) chose the rewarded response significantly more often than the neutral response indicating that they successfully learned the response–outcome associations (Figure 2A; Table 2). This learning effect can be considered as strong (*p* < 0.001, *d* = 2.9). Weight category did not significantly influence the number of rewarded and neutral or the total number of responses in instrumental conditioning (Table 2). Participants neutral key presses still make up approximately 25% of all responses, which is probably due to the partial reinforcement schedule applied during the instrumental task.

**Table 2:** Statistical analysis of the instrumental conditioning.

Effect	df	*F* (nr responses)	*p* (nr responses)
Response type	1, 51	117.6	0.001*
Weight category	2, 51	0.4	0.698
Response type*weight category	2, 51	0.2	0.824

*Response type defines if a rewarded or neutral response was made*.

*Asterisks indicate significant effects*.

### Pavlovian Conditioning Task

Our analysis of eye movements indicated that all participants (*N* = 54) successfully learned the stimulus–outcome associations during Pavlovian conditioning. Specifically, we analyzed the participants eye movements after stimulus onset before the outcome was displayed (i.e., during the neutral screen, see Figure 1B).

The eye index was analyzed in bins of five trials to capture learning effects for the rewarded and neutral condition (Figure 2B) and each weight category (Figures 2C–E; Table 3). The rewarded condition showed a significantly higher eye index than the neutral condition (*p* < 0.001, *d* = 0.41, Figure 2B). This finding indicates that for the rewarded condition and throughout the conditioning task, participants spent more time fixating the reward than the cue location. This was different from the neutral condition, in which participants spent relatively more time fixating the cue location.

**Table 3:** Statistical analysis of eye index during the Pavlovian conditioning.

Effect	df	*F* (eye index)	*p* (eye index)
Condition	1, 561	51.9	0.001*
Time	5, 561	0.5	0.747
Weight category	2, 51	0.3	0.738
Condition*time	5, 561	0.5	0.794
Condition*weight category	2, 561	2.3	0.107
Time*weight category	10, 561	1.0	0.453
Condition*time*weight category	10, 561	1.9	0.042*

*Asterisks indicate significant effects*.

We found a significant interaction between condition, time and weight category (*p* < 0.05, Figures 2C–E; Table 3). This effect was driven by patterns of fixation by condition and time in each of the three weight groups. Normal-weight participants consistently fixated on the reward location for rewarded cues and the cue location for neutral cues after the first time-bin. In contrast, overweight participants fixated primarily on the reward location irrespective of whether they saw the rewarded or the neutral cue and this fixation pattern was stable over time. Obese participants showed yet another fixation pattern in that they immediately favored the reward location for the rewarded cues and initially favored the cue location on neutral trials. However, in the second half of the trials the obese subjects shifted to favoring the reward location for neutral cues as well.

In a control analysis, we analyzed the percentage of time participants spent looking at other areas than the defined area of interest (i.e., upper and lower square) for the first and second half of the trials in each condition (Table 4). Participants spent slightly more time outside the area of interest after the neutral compared to the rewarded stimulus (reward = 19.13 ± 15.58, neutral = 22.85 ± 15.72, *p* < 0.001, *d* = −0.24). Furthermore, participants spent slightly more time outside the area of interest in the second compared to the first half of the experiment (first = 19.85 ± 15.20, second = 22.13 ± 16.23, *p* < 0.05, *d* = −0.15). In addition, the percentage of time where eye movements could not be tracked for example because of blinks or not focusing the screen (i.e., missing values) changed significantly over time (first = 7.58 ± 11.39, second = 10.79 ± 14.66, *d* = −0.24, *p* < 0.001) and it was slightly higher after the neutral cue (reward = 8.60 ± 12.58, neutral = 9.76 ± 13.82, *p* = 0.090) (Table 4). Approximately 9% of the eye-tracking data was discarded from the analysis. Importantly, weight category had no significant influence on the time spent outside of the target areas or on missing values where eye tracking failed.

**Table 4:** Statistical analysis of the time participants spent outside the targets and missing values during Pavlovian conditioning.

Effect	df	*F* (time outside)	*p* (time outside)	*F* (not tracked)	*p* (not tracked)
Condition	1, 153	13.2	0.000*	2.9	0.090
Time	1, 153	4.7	0.032*	20.2	0.001*
Weight category	2, 51	0.9	0.407	1.2	0.318
Condition*time	1, 153	3.0	0.086	0.1	0.782
Condition*weight category	2, 153	0.1	0.869	0.8	0.439
Time*weight category	2, 153	0.3	0.728	0.3	0.723
Condition*time*weight category	2, 153	1.7	0.185	0.6	0.528

*Asterisks indicate significant effects*.

### PIT Task

To test for a PIT effect and possible differences between weight categories and fixation style measured during Pavlovian conditioning, we added these factors as between-subject factors to a linear mixed-effects model. Weight categories were formed based on BMI and fixation style based on a median split on the conditioned eye response to the rewarded cue in the second half of the Pavlovian conditioning (see Analysis, for more detail). Furthermore, we added impulsiveness (BIS) and depression (BDI) total scores as covariates of no interest to our statistical model of PIT. This was based on previous literature, which has shown that the PIT effect may be influenced by depression and that the conditioned response is associated with impulsiveness (49, 52, 85–87).

**Figure 3:**
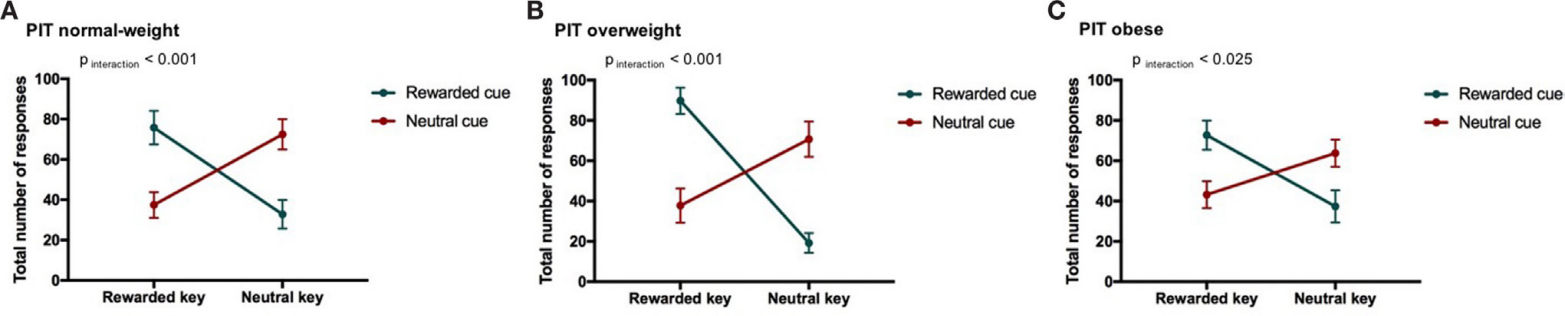
Result from Pavlovian-to-instrumental transfer (PIT) and weight category. Error bars indicate SEM. The rewarded condition is depicted in green and the neutral condition in red. The strength of the PIT effect depends on weight category (normal-weight *N* = 20, overweight *N* = 17, obese *N* = 17, *p*_CONDITION*RESPONSE TYPE*WEIGHT CATEGORY_ < 0.001). The goal-directed behavior of overweight individuals was most strongly influenced by cues. **(A)** PIT normal-weight, **(B)** PIT overweight, and **(C)** PIT obese.

We found a PIT effect such that participants chose the rewarded response more often than the neutral response when the rewarded cue was displayed and vice versa for the neutral cue. The strength of the PIT effect was modulated depending on the participant’s weight status as indicated by a significant CONDITION*RESPONSE TYPE*WEIGHT CATEGORY effect (*p* < 0.001, Tables 5 and 6; Figure 3). This effect reflects that the PIT effect was strongest in overweight individuals (Figure 3B, *p*_CONDITION*RESPONSE IN OVERWEIGHT_ < 0.001), which were highly sensitive to the presence of the rewarded cue (causing a clear preference for selecting the rewarded key). The PIT effect in normal-weight and obese participants was also present but clearly smaller (*p*_CONDITION*RESPONSE IN NORMAL-WEIGHT_ < 0.001, *p*_CONDITION*RESPONSE IN OBESE_ < 0.025). Participants pressed the neutral key also during the rewarded cue presentation presumably due to the partial reinforcement schedule used in the instrumental conditioning task.

**Table 5:** Statistical analysis of the number of responses during the Pavlovian-to-instrumental transfer including the repeated factors CONDITION, RESPONSE STYLE, and the group variable WEIGHT CATEGORY.

Effect	df	*F* (nr responses)	*p* (nr responses)
Condition	1, 108	1.5	0.229
Response type	1, 108	0.1	0.929
Weight category	2, 36	3.8	0.031*
Condition*response type	1, 108	0.8	0.370
Condition*weight category	2, 108	0.3	0.742
Response type*weight category	2, 108	0.1	0.999
Condition*response type*weight category	2, 108	3.4	0.036*

*Asterisks indicate significant effects*.

**Table 6:** Statistical analysis of the number of responses during the Pavlovian-to-instrumental transfer including the repeated factors CONDITION, RESPONSE STYLE, and the group variables WEIGHT CATEGORY, FIXATION STYLE.

Effect	df	*F* (nr responses)	*p* (nr responses)
Condition	1, 72	0.1	0.934
Response type	1, 72	0.9	0.357
Weight category	2, 24	2.7	0.090
Fixation style	1, 24	1.1	0.309
Condition*response type	1, 72	0.1	0.851
Condition*weight category	2, 72	0.1	1.000
Condition*fixation style	1, 72	0.1	0.955
Response type*weight category	2, 72	0.2	0.786
Response type*fixation style	1, 72	0.1	0.786
Weight category*fixation style	2, 24	0.1	0.869
Condition*response type*weight category	2, 72	8.9	0.001*
Condition*response type*fixation style	1, 72	0.6	0.454
Condition*weight category*fixation style	2, 72	0.2	0.999
Response type*weight category*fixation style	2, 72	0.1	0.872
Condition*response type*weight category*fixation style	2, 72	4.0	0.022*

*Asterisks indicate significant effects*.

We also reported a significant main effect of WEIGHT CATEGORY (*p*_WEIGHT CATEGORY_ < 0.05, Table 5). However, the differences in the total number of responses between weight categories were in a very small range (normal-weight = 57 ± 38, overweight = 55 ± 41, obese = 54 ± 32). Therefore, we do not believe that this represents a general difference in motivation to do the task.

**Figure 4:**
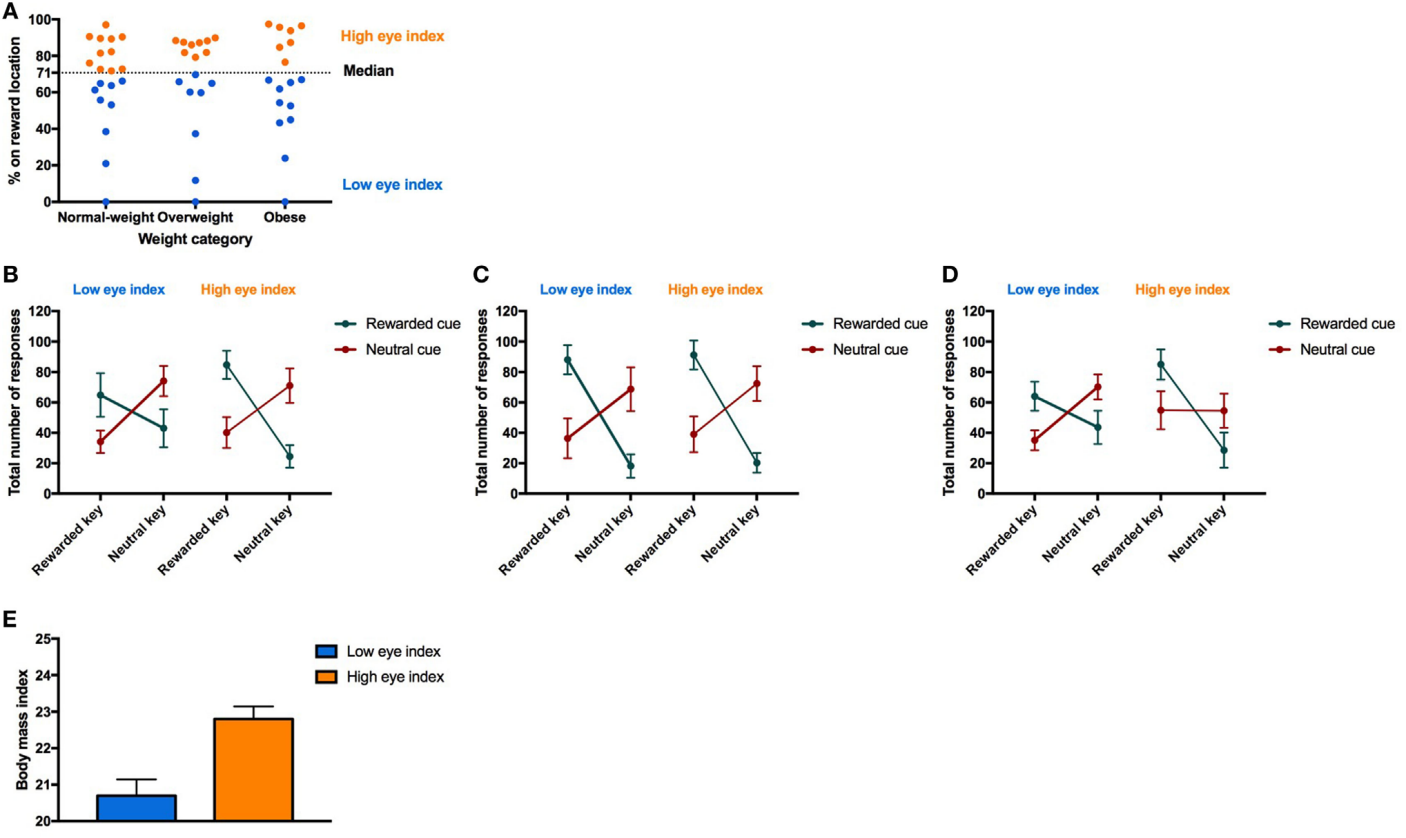
Result from Pavlovian-to-instrumental transfer (PIT) for the “low eye index” and “high eye index” group. Error bars indicate SEM. The rewarded key/condition is depicted in green and the neutral key/condition in red. The “low eye index” group is represented in blue and the “high eye index” group in orange. **(A)** Categorization of fixation style. Fixation style was based on a median split on the conditioned eye response in the second half of all trials after the rewarded cue during Pavlovian conditioning. **(B–D)** PIT effect for different weight categories and fixation styles. Fixation style showed a significant influence on PIT depending on the weight category (*p*_CONDITION*RESPONSE TYPE*WEIGHT CATEGORY*FIXATION STYLE_ < 0.025). In the normal-weight group, the “low eye index” group consisted of 11 and the “high eye index” of nine individuals. In the overweight group, the “low eye index” group consisted of eight and the “high eye index” of nine individuals. In the obese group, the “low eye index” group consisted of 10 and the “high eye index” of 7 individuals. **(E)** Body mass index (BMI) in normal-weight individuals for each fixation style. The “high eye index” group showed an increased BMI within the healthy range compared to the “low eye index” group (*p* < 0.001, *d* = 1.7).

Next, we tested the association between the conditioned response behavior measured during Pavlovian conditioning (i.e., fixation style) and the PIT effect. Therefore, we identified two groups “low eye index” (i.e., individuals who preferentially fixated the cue location) versus “high eye index” (i.e., individuals who preferentially fixated the reward location) which were similarly distributed across the weight categories (Figure 4A). Statistics revealed that the PIT effect is modulated by fixation style but that this modulatory effect depends additionally on weight category (four-way interaction CONDITION*RESPONSE TYPE*WEIGHT CATEGORY*FIXATION STYLE, Table 6; Figures 4B–D). In both the normal-weight (Figure 4B) and obese groups (Figure 4C), individuals showing a high eye index exhibited a stronger PIT effect triggered by reward cues than individuals showing a low eye index. By contrast, in overweight participants this dissociation was absent, i.e., we observed a high PIT effect irrespective of whether individuals exhibited low or high eye index tendencies during conditioning. Interestingly, obese individuals with a high eye index (Figure 4D) were not only sensitive to the reward cue but also largely *insensitive* to the neutral cue since they chose the congruent versus incongruent key with nearly equal probability for this latter condition.

Finally, we tested whether there is an association between the fixation style observed during Pavlovian conditioning and BMI by running separate mixed-effect models within each of the weight groups. Unexpectedly, we found that normal-weight individuals of the “high eye index” group showed an increased BMI within the healthy range (*d* = 1.7, *p* < 0.001, Figure 4E). This effect was surprisingly strong and was not be found in overweight or obese individuals (*p* > 0.646).

## Discussion

Here, we tested whether sensitivity to rewards and reward-predicting cues is abnormal in overweight and obese individuals versus normal-weight controls and whether such differences in reward sensitivity modulate goal-directed behavior. We addressed this question with a PIT experiment and investigated whether food-predicting cues differentially influence goal-directed behavior of normal-weight, overweight, and obese individuals. Furthermore, we applied eye tracking during Pavlovian conditioning as a proxy of the incentive salience of the predicted reward. Our findings imply that cue-controlled behavior might be altered in overweight and obese individuals as discussed in further detail below.

### Overweight Participants Exhibit a Higher PIT Effect than Normal-Weight or Obese Individuals

Overweight participants showed the strongest PIT effect compared to normal-weight and obese subjects (see PIT Task, Figures 3A–C). This finding extends previous observations that overweight and obese adults showed enhanced reactivity to food stimuli during the passive observation of stimuli, a visual dot probe task, different versions of the Stroop task or in questionnaires (37, 87, 89). These studies quantified food cue reactivity by measuring reaction time, eye-tracking duration and direction biases, pupil diameter, electroencephalography, and functional magnetic resonance imaging (37). Eye tracking in particular revealed duration orienting biases toward food cues and decreases in pupil diameter [a marker of noradrenergic increases and higher attentional engagement (90, 91)] to high-calorie foods in overweight and obese subjects (92–94). Our results extend these previous reports by showing that goal-directed behavior in overweight individuals is strongly influenced by cues associated with food rewards, as tested by the PIT paradigm, while the influence of neutral cues was similar to the normal-weight group. Interestingly, no such reward-specific PIT effect was observed for the group of obese individuals. Note that there were no group differences in food liking. Even though this result in obese individuals is puzzling at first, it is in line with a recent study which also found that obese individuals had a PIT effect comparable to normal-weight subjects (57). However, Watson et al. (57) showed an increased PIT effect for high-calorie versus low-calorie foods, which was only found in obese subjects (57). One potential explanation of the finding that the PIT effect is similar in obese and healthy individuals is that habitual intake of energy dense diets may induce a compulsive style of eating that is insensitive to environmental cues (see Physiological Mechanism and Open Questions).

Taken together, our finding that motivation induced by reward-related cues is increased in overweight individuals is in line with the incentive sensitization theory of addiction (15–21). The incentive sensitization theory of addiction predicts an attentional bias toward reward-related cues, which is in line with our eye movement results during Pavlovian conditioning, and a pathological motivation for rewards and reward-related cues (i.e., compulsive “wanting”) (17, 20). The pathological motivation for food and food-predicting cues was in the present study displayed by the increased PIT effect in overweight individuals. Some studies in humans investigating the influence of Pavlovian cues on instrumental responding in substance dependence also showed an increased PIT effect in addicts compared to controls (48, 57, 95). There is nevertheless some evidence for no association between PIT and substance dependence in other studies (11, 42, 43, 50, 56, 96).

However, our data further indicate that once the obese status is reached, incentive sensitization might return to normal levels. It is tempting to speculate that hypersensitivity might be reduced in obese individuals due to habitual/compulsive overeating (97, 98), but this was not directly tested in the present study. It is also possible that obese individuals may direct less attention toward small food rewards (as used here) and/or their preference may be shifted to stimuli with a larger subjective value (e.g., more palatable and calorie rich rewards), which has been shown to significantly influence PIT (53). We did not collect data on the subjective reward value in the present study. Therefore, possible differences in reward valuation between weight groups might offer an alternative explanation for the reduced PIT effect observed in obese individuals.

### Eye Movements during Pavlovian Conditioning Differ between Normal-Weight, Overweight, and Obese Individuals

We employed eye tracking to measure behavioral changes during Pavlovian conditioning. Eye tracking has been used previously to measure reactivity to passively observed food stimuli (34, 37) and to investigate individual differences in the extent to which individuals attribute incentive salience to reward-predicting cues versus the reward itself (49). Here, we performed eye tracking in the period between seeing the cue and receiving a reward, i.e., while participants saw only a neutral screen but no visual stimuli. We chose to modify previous paradigms (49) because gaze is automatically attracted to visual cues unless these eye movements are actively inhibited.

In our study, the conditioned eye response toward the rewarded and neutral cue location during Pavlovian conditioning was differently modulated depending on the participant’s weight status (see Pavlovian Conditioning Task, Figures 2C–E). More specifically, we found during Pavlovian conditioning that overweight individuals exhibited a general orientation bias toward the reward location irrespective of whether they performed a reward cue trial or a neutral cue trial. This lack of a clear dissociation between reward and neutral trials remained relatively stable across conditions and is broadly consistent with the observation that overweight adults showed enhanced reactivity to food stimuli during the passive observation of stimuli, a visual dot probe task, different versions of the Stroop task or in questionnaires (37, 87, 89). Specifically, we confirmed and extend these studies by showing that overweight individuals exhibit a general duration orientation bias toward the reward location, suggesting larger sensitivity to the anticipated reward, an interpretation that is consistent with a larger PIT effect for cues that have been associated with food rewards. Also, obese individuals differed from normal-weight controls but mainly during the initial half of Pavlovian conditioning, where they exhibited a clear distinction between conditioned responses to reward cues (which caused long fixation durations on the reward location) and the neutral cues (which resulted in longer fixation durations of the cue location). However, this strong initial differentiation was clearly reduced at the end of the Pavlovian conditioning.

### Individual Differences in Conditioned Responses Differentially Influence PIT Effects in Normal-Weight, Overweight, and Obese Individuals

We used the eye movement behavior to detect individual differences and categorize the participants into a group of individuals with a “low eye index,” i.e., they fixated predominantly the cue location or “high eye index,” i.e., they fixated predominantly the reward location. Our experiment revealed that normal-weight individuals of the group “high eye index” showed a stronger PIT effect for the reward cue than individuals of the group “low eye index” (Figures 4B,E). There is only one group of researchers that performed a similar experiment to investigate the influence of the individual fixation style on PIT (49). Contrary to our results, they found that a stronger conditioned eye movement response toward the cue led to an increased modulation of goal-directed behavior. However, they quantified eye movements while the cue was still on the screen proposing that the eye movement behavior was a proxy of cue approach behavior observed in animals, also known as “sign-tracking” (22, 23, 58, 99, 100). By contrast, we tested the conditioned eye response during a neutral screen suggesting that the eye movement behavior might mainly reflect the incentive salience of the predicted reward (see Figure 1B). We found that individual differences during Pavlovian conditioning (i.e., “low” versus “high eye index”) interacted with the weight category to influence PIT.

In both the normal-weight and obese groups, the “high eye index” group exhibited a stronger PIT effect triggered by reward cues than the “low eye index” group. By contrast, in overweight participants we observed a high PIT effect irrespective of whether individuals exhibited high or low eye index tendencies during conditioning. However, these data have to be interpreted with caution because the subgroups were quite small. One possible explanation for individual differences in the PIT effect is that not only incentive salience, but also inhibitory control has an impact on how goal-directed behavior is influenced by Pavlovian cues. Normal-weight and obese individuals expressing a “low eye index” might show a smaller PIT effect because they express an inhibitory control mechanism, which regulates the influence of reward-related cues on goal-directed behavior. However, in overweight expressing a “low eye index” this inhibitory mechanism might be altered so that they express a stronger PIT effect, which means that these participants are more susceptible to the influence of cues. Response inhibition for example with a Go/Nogo task was not tested in the present study. Nevertheless, reduced response inhibition was previously shown to be related with overeating and unsuccessful dieting (101, 102). Our finding is also in line with Trick et al. (103) who have shown that a higher conditioned response measured during Pavlovian conditioning is not automatically translated into a higher PIT. The same applies to electrophysiological responses (i.e., P300) that were not correlated with the PIT effect in social drinkers (96).

Furthermore, we found that normal-weight expressing a “high eye index” showed an increased BMI within the healthy range. This could be linked to previous research suggesting that an increased attentional bias toward food cues as a risk factor for gaining weight (37). However, a recent review of the literature has shown that attention to food or drug cues is a weak index of the problem behavior (104).

### Interpretational Issues

Our research paper presents a novel view on how food-related cues influence eye movements and goal-directed behavior in overweight and obese individuals. However, the interpretation of our findings is subject to specific limitations.

First, individual differences in reward valuation could have influenced cue-controlled behavior. We tried to overcome this issue by testing all participants in the same dietary state (i.e., hungry) and by letting them choose their favorite snack out of four options. Reward liking based on a visual analog scale was not different between groups (WEIGHT CATEGORY, FIXATION STYLE) and has not influenced the conditioned eye response nor PIT.

Second, our experiment does not enable us to determine whether the overweight individuals’ sensitivity to environmental cues holds only for food-specific cues or whether these individuals show a generally increased sensitivity to reward-predicting cues. Both general and substance-specific effects of reward have been found in previous studies on alcohol-dependent patients (45, 48) and smokers (95). Thus, although the dissociation of general and food-specific reward effects was not the focus of the present study, it represents an important question for future research.

### Physiological Mechanism and Open Questions

What exactly might be the underlying mechanism for finding differences in the conditioned eye response and probably also the goal-directed behavior in normal-weight, overweight, and obese individuals? It is well-established that eating palatable food increases brain activity in regions implicated in reward processing (i.e., striatum, midbrain, amygdala, orbitofrontal cortex) and leads to a dopamine release in the dorsal striatum. The amount of dopamine is related to the pleasantness ratings (i.e., “liking”) and the caloric density of the reward/food [for reviews, see Ref. (20, 21)]. Anticipated food intake or exposure to cues/food images increases activity within brain regions known for incentive reward valuation (i.e., amygdala, orbitofrontal cortex) (21, 105, 106) and results in a similar dopamine release as rewards (107). The incentive sensitization model posits that repeated intake of high-calorie palatable food leads to an increased brain activity in regions involved in incentive valuation to cues that are associated with palatable food intake *via* conditioning, which prompts craving and overeating when these cues are available (15, 17, 20, 21). There is strong evidence that dopaminergic neurons projecting to the striatum and ventral pallidum respond to the receipt of palatable food, but after repeated pairings between food and a cue, fire in response to the food-related cue and no longer in response to the receipt of food [for review, see Ref. (107)]. This shift during stimulus-outcome learning attributes value to the cues themselves and thereby guides motivated behavior (59, 107–109). This process is likely to contribute to overeating and lead to weight gain. Consistent with the incentive sensitization theory, obese humans showed an increased activity in brain regions associated with reward and motivation, brain regions associated with motor responses and brain regions associated with attention to food pictures, food cues, or food commercials (20, 21, 27, 110–114). This greater responsivity to food-associated cues could be reflected in the increased conditioned eye response in obese individuals observed in our experiment. A food-related cue attributed with incentive salience can then trigger actions to obtain the food (i.e., increased “wanting”) (20). In our study, this increased “wanting”/motivation due to food-associated cues is a potential reason for observing stronger PIT effects in overweight. However, our study suggests that this is probably not the case for obese participants. There is some evidence from animal and human experiments that habitual intake of high-fat diets decreases dopamine signaling in the reward circuitry (21, 115, 116). This is in agreement with experiments on cocaine and alcohol-dependent individuals (117, 118). However, habitual processes were not measured with our experimental paradigm.

A combination of our behavioral paradigm with additional methods such as neuroimaging or pharmacological interventions would allow better understanding of the underlying mechanism. This would also facilitate the integration of our findings into animal research on individual variation, conditioned motivation, overeating, and addiction. Furthermore, it would be interesting to investigate the influence of environmental cues in a group of patients after bariatric surgery or after other interventions (i.e., diet, behavioral training, see Clinical Implication).

### Clinical Implication

Our findings may prove to be of practical relevance because we show that the overweight group’s conditioned eye response and goal-directed behavior is generally more susceptible to the influence of environmental cues. Thus, it might be beneficial to address mental strategies to resist food-related cues also in the psychological/behavioral treatment of overweight individuals [e.g., extinction training, attentional control training, response training (60, 119–121)]. Manipulating the attentional bias to drug cues *via* attentional control therapies was shown to reduce some of the behavioral control drug cues have over addicts (60, 122–124). To the best of our knowledge, there is only one study, which applied the attention bias modification (ABM) program as used in addictive disorders to overweight and obese individuals (i.e., binge eaters) (125). This study revealed a decrease in weight, eating disorder symptoms, binge eating, and loss of control and responsivity to food after an 8-week ABM training (125). However, these results should be interpreted with caution because of the low sample size and single-group open label trial. A combination of food response and attention training has successfully downregulated reward and attention brain networks and reduced body fat (120, 121). For obese individuals, which in our study did not differ from normal-weight controls regarding the influence of external cues on goal-directed behavior, other treatments are possibly more appropriate because maladaptive eating behavior has already been consolidated [e.g., cognitive behavioral therapy, motivational interviewing, habit reversal training, inhibition control training (102, 126)]. The finding of the present study together with previous studies (8, 9, 14, 57) should also be considered when new policies and guidelines for food advertisements will be drafted.

## Conclusion

We found that PIT effects for food rewards differed as a function of weight status. In particular, overweight individuals were more strongly influenced by food-associated stimuli than both obese and normal-weight individuals. Eye movements during Pavlovian conditioning were not related to the strength of the PIT effect in overweight or obese individuals. However, normal-weight individuals with a stronger conditioned response toward the reward location showed a stronger PIT effect and are possibly at risk to gain weight. Our findings are generally in line with the incentive sensitization theory predicting that overweight individuals are more susceptible to food-related cues than normal-weight controls. We speculate that this hypersensitivity might be reduced in obese participants due to habitual/compulsive overeating or differences in reward valuation.

## Ethics Statement

All subjects gave written informed consent in accordance with the Declaration of Helsinki. The protocol was approved by the Ethics Committee of the Canton Zurich.

## Author Contributions

All authors conceived of and designed the experiment; RL programmed the experiment, analyzed the data, wrote the main manuscript text, and prepared the figures; AB collected the data; all authors read, corrected, and approved the final manuscript.

## Conflict of Interest Statement

The authors declare that the research was conducted in the absence of any commercial or financial relationships that could be construed as a potential conflict of interest.
